# Analysis of variants in DNA damage signalling genes in bladder cancer

**DOI:** 10.1186/1471-2350-9-69

**Published:** 2008-07-18

**Authors:** Ananya Choudhury, Faye Elliott, Mark M Iles, Michael Churchman, Robert G Bristow, D Timothy Bishop, Anne E Kiltie

**Affiliations:** 1 Cancer Research UK Clinical Centre, Section of Oncology, Leeds Institute of Molecular Medicine, Leeds, LS9 7TF, UK; 2 Section of Epidemiology and Biostatistics, Leeds Institute of Molecular Medicine, University of Leeds, Leeds, LS9 7TF, UK; 3 CR-UK Genotyping Facility, University of Leeds, St. James's University Hospital, Leeds, LS9 7TF, UK; 4 Ontario Cancer Institute, Princess Margaret Hospital, Toronto, M5G 2M9, Canada

## Abstract

**Background:**

Chemicals from occupational exposure and components of cigarette smoke can cause DNA damage in bladder urothelium. Failure to repair DNA damage by DNA repair proteins may result in mutations leading to genetic instability and the development of bladder cancer. Immunohistochemistry studies have shown DNA damage signal activation in precancerous bladder lesions which is lost on progression, suggesting that the damage signalling mechanism acts as a brake to further tumorigenesis. Single nucleotide polymorphisms (SNPs) in DSB signalling genes may alter protein function. We hypothesized that SNPs in DSB signalling genes may modulate predisposition to bladder cancer and influence the effects of environmental exposures.

**Methods:**

We recruited 771 cases and 800 controls (573 hospital-based and 227 population-based from a previous case-control study) and interviewed them regarding their smoking habits and occupational history. DNA was extracted from a peripheral blood sample and genotyping of 24 SNPs in *MRE11, NBS1, RAD50, H2AX *and *ATM *was undertaken using an allelic discrimination method (Taqman).

**Results:**

Smoking and occupational dye exposure were strongly associated with bladder cancer risk. Using logistic regression adjusting for age, sex, smoking and occupational dye exposure, there was a marginal increase in risk of bladder cancer for an *MRE11 *3'UTR SNP (rs2155209, adjusted odds ratio 1.54 95% CI (1.13–2.08, p = 0.01) for individuals homozygous for the rare allele compared to those carrying the common homozygous or heterozygous genotype). However, in the hospital-based controls, the genotype distribution for this SNP deviated from Hardy-Weinberg equilibrium. None of the other SNPs showed an association with bladder cancer and we did not find any significant interaction between any of these polymorphisms and exposure to smoking or dye exposure.

**Conclusion:**

Apart from a possible effect for one MRE11 3'UTR SNP, our study does not support the hypothesis that SNPs in DSB signaling genes modulate predisposition to bladder cancer.

## Background

Tobacco smoke and occupational carcinogens are the major risk factors for urothelial cell carcinoma of the bladder. Products in cigarette smoke cause oxidative DNA damage which is repaired by base excision repair (BER). Bulky adducts from metabolism of polycyclic aromatic hydrocarbons and aromatic amines [1] are repaired by nucleotide excision repair (NER), although other damage requires other pathways [2-4]. The most lethal form of DNA damage is the DNA double strand break (DSB) which if not repaired can lead to cell death [5]. DSB can be produced by oxidative lesions in close proximity on opposing DNA strands or during repair of bulky adducts causing interstrand cross links which requires a combination of NER and homologous recombination for their repair. As only a small proportion of individuals exposed to environmental carcinogens develop bladder cancer, it has been suggested that genetic factors are important in determining the response to carcinogen exposure [6].

Cell-cycle checkpoints and DNA damage repair are two mechanisms which protect the cell against genetic instability and mutagenesis [7]. The ATM, H2AX, Chk2 and p53 proteins are involved in DNA damage recognition and consequent cell cycle arrest allowing DNA repair or, if repair fails, cell death.

Other proteins involved in signalling of DSB damage include the MRE11-RAD50-NBS1 (MRN) complex which has been shown to act both upstream of ATM, with NBS1 responsible for the activation of ATM, and downstream of ATM, leading to the activation of DSB repair by homologous recombination or non-homologous end joining. DSB are also formed during mitosis when replication forks arrest and the MRN complex has also been implicated in the signalling pathway for the detection of these collapsed replication forks [8]. The MRN complex is involved in G1/S cell cycle checkpoint activation and can phosphorylate Chk2 [9], while Chk1, involved in the G2/M checkpoint, is phosphorylated by ATM or ATR in response to DNA damage [10,11]. Telomere integrity is important for genomic stability, and cells deficient in ATM or MRE11 have shortened telomeres [12]. ATM and the MRN complex are thought to be involved in telomere stabilization by preventing fusion between the free ends of the chromosomes [13]. H2AX is rapidly phosphorylated at the sites of DSB and is important for the recruitment of repair proteins [14]. Interestingly, *MRE11, ATM *and *H2AX *are located on the long arm of chromosome 11. *MRE11 *is located at 11q21, *ATM *at 11q22.3 and *H2AX *at 11q23.2-23.3.

Compared to the small proportion of cancers associated with high penetrance mutations, the majority of cancers are thought to be caused by a combination of low penetrance genes and environmental factors. Single nucleotide polymorphisms (SNPs) are found in numerous DNA repair genes in the general population. Individuals vary markedly in their intrinsic DNA repair capacity and there is evidence that decreased repair capacity is associated with increased cancer risk [6,15]. SNPs in the DNA repair signalling genes may account for some of this variation [16].

A number of studies have looked at variants in genes in the various DNA repair pathways, mainly focusing on the BER and NER pathways, and also cell cycle genes [6,17-20,23-25] and association with bladder cancer. No single variant has been conclusively associated with bladder cancer risk. In a large case-control study conducted by Garcia-Closas et al [17], variant genotypes of SNPs within the NER pathway genes were found to be associated with small increases in bladder cancer risk (with odds ratios ranging between 1.2 and 1.4). Wu et al [6] studied 44 SNPs in 33 genes associated with DNA repair and cell cycle control. They found that only three of the SNPs, *XPD *Asp312Asn, *RAG1 *Lys820Arg, and a *TP53 *intronic SNP exhibited statistically significant effects, but that increasing numbers of potentially high risk alleles within the NER pathway or the combined DNA repair and cell cycle control pathways had a significant effect on increased bladder cancer risk. We have previously shown an association between three SNPs in *XPC*, one of the key NER genes, and bladder cancer risk [18]. Sanyal et al [19] studied a number of SNPs in DNA repair genes in 327 bladder cancer patients including one in *NBS1 *(Glu185Gln), but this was not found to be significantly associated with bladder cancer risk. Figueroa et al recently found no association with four *NBS1 *variants and bladder cancer risk [26]. However, variants in other components of the DSB signalling pathway have not yet been studied in bladder cancer.

Large case-control studies in breast cancer have not shown any association between variants within the *ATM *and *NBS1 *genes and disease risk [27-32], although *ATM *variants have been weakly associated with an increased risk of lung cancer in two studies [33,34], and *NBS1 *Glu185Gln has been associated with increased lung cancer risk in a Chinese study [35], but not in a Norwegian study [36].

We hypothesized that potentially functional SNPs within the *MRE11, RAD50, NBS1, ATM *and *H2AX *genes, by affecting DSB signalling and genomic stability may modulate predisposition to bladder cancer, and that these SNPs may modify the bladder cancer risk associated with smoking and occupational exposures.

## Methods

### Cases and Controls Selection and Recruitment

This has been described previously [20]. Ethical approval was obtained from Leeds (East) Local Research Ethics Committee (LREC). Patients with histologically proven bladder cancer were recruited at the Pyrah Department of Urology, St James's University Hospital, Leeds, (SJUH) from August 2002 to March 2006.

Five hundred and seventy-three control individuals were recruited from the ophthalmology and ear, nose and throat (ENT) departments, SJUH (hospital-based controls). Informed consent was obtained from each subject. Attempts were made to frequency match the control population for sex as bladder cancer is more prevalent in males. Individuals had no previous history of bladder cancer or symptoms of haematuria. A second group of 227 controls were used from a previous case-control study undertaken by the Genetic Epidemiology Laboratory, CR-UK Clinical Centre, Leeds (community controls) [37].

Information regarding smoking habits, occupation (exposure to occupational carcinogens) and ethnicity (Caucasian or non-Caucasian) were obtained from direct interviews with both case and control subjects.

### DNA Extraction and Storage

Five millilitre blood samples were obtained from cases and controls. All blood samples were sent to the Regional Genetics Laboratory, SJUH, for DNA extraction using a salt precipitation method and stored at -20°C until required as previously described [20].

### Selection of SNPs for MRE11, NBS1, RAD50, ATM and H_2_AX

A list of SNPs for *MRE11, NBS1, RAD50, ATM *and *H2AX *were obtained from the public domain of the Environmental Genome Project (EGP) via the National Center for Biotechnology Information (NCBI)  and GeneSNP from the University of Utah . Non-synonymous exonic SNPs, SNPs at known splice sites within 50 base pairs downstream and upstream of the exon, and 5' and 3' UTR SNPs with an allele frequency >3% were chosen. Although 30 SNPs were selected, six could not be genotyped using the TaqMan method (Applied Biosystems, Foster City, CA). A total of 24 SNPs were genotyped.

### Genotyping

The DNA samples were sent for high throughput genotyping to the CR-UK Genotyping Facility in Oxford using the TaqMan method[20]. To ensure quality control, the DNA was viewed on an agarose gel to check molecular weight and look for fragmentation or degradation. All DNA was quantified using pico green and then normalised to 50 ng/ul before being diluted to 5 ng/ul. A PCR reaction for a β-Actin fragment (approx 500 bp) was performed to check the quality of DNA. On the TaqMan plates, non-template controls were included (blanks) to indicate the background fluorescence and, hence, illustrate positives from failures. Commercially purchased DNA was included on all plates (positive controls) to ensure that each plate had amplified successfully. Five percent of samples were blind duplicates so that concordance between genotype calls could be assessed. Genotyping calls were checked by two people independently.

### Confirmatory sequencing of *MRE11 *rs2155209

Confirmatory sequencing was performed for the *MRE11 *variant rs2155209. Thirty two wild type, 31 heterozygous and 32 variant homozygous genotyped samples were identified across the 96-well plates and the samples sequenced using a Big Dye Terminator v1.1 Cycle Sequencing kit as per the manufacturer's instructions. Briefly, the region containing the variant was amplified by PCR reaction in a thermal cycler using the following primer sequences: Forward: 5' GGCTAATTATGGTATTACTGCATAGG 3', Reverse: 5' TCAAGCATTTAGGAATGTGACC 3'. PCR products were cleaned up with ExoSap-IT (GE Healthcare, Little Chalfont, UK) and then directly sequenced in a reaction mix containing v1.1 BigDye Terminator reaction mix (Applied Biosystems, Warrington, UK). DNA sequencing products were cleaned up by ethanol precipitation, resuspended in Hi-Di formamide (Applied Biosystems, Warrington, UK) and analysed on an Applied Biosystems 3730 × l DNA analyser. Sequencing data was analysed using Mutation Surveyor software (Softgenetics, Pennsylvania, USA).

### Statistical Analysis

All statistical analysis was undertaken using STATA9 software (StataCorp, Texas, USA). The genotype frequency of each SNP was tested for deviation from Hardy-Weinberg equilibrium amongst the controls. This was done by comparing the observed genotype frequencies with the expected frequencies using a Chi-squared test. Minor allele frequencies for each SNP were compared to those in the NCBI database. Pairwise Lewontin's D' was calculated to determine linkage disequilibrium between the SNPs.

Pearson's Chi-squared tests were used to compare sex and ethnicity between cases and controls and a Two-tailed T-test was used for age at diagnosis for cases and age at blood sampling for the controls. Smoking status was categorized as ever versus never smoked. Pack years of smoking was also calculated (number of cigarettes per day/20) × number of years smoked)). Exposure to six occupational hazards (rubber, plastics, labs, printing, dyes, diesel) were analysed as ever versus never and the total number of exposures was calculated from these six occupational hazards. Odds ratios and 95% confidence intervals were estimated for each occupational hazard and smoking status (ever versus never) separately on bladder cancer risk. Smoking status (ever versus never) and occupational dye exposure (ever versus never) were then entered into a logistic regression model together to assess the independence of these two risk factors on bladder cancer. The analyses for smoking status (ever versus never) and dye exposure (ever versus never) were then repeated, stratified by genotype group for each SNP to assess potential differential effects of smoking and dye exposure separately by genotype group. Likelihood ratio tests were carried out to test for gene-exposure interactions by comparing a model including an interaction term to a model including only the main effects.

Odds ratios and 95% confidence intervals were estimated to assess the effect of each SNP on bladder cancer risk unadjusted and then adjusted for age, sex, smoking (ever versus never) and dye exposure (ever versus never) in multivariable logistic regression. Simhap (McCaskie, 2004) [21] was used to calculate haplotype odds ratios and 95% confidence intervals from logistic regression, after using estimation maximisation techniques to infer haplotypes for the unphased genotype data.

In order to determine if multiple SNPs within the pathway may have an additive effect on bladder cancer risk, a combined analysis of four SNPs, one from each of *NBS1, MRE11, ATM *and *H2AX *was undertaken. A SNP from each gene was chosen by picking the SNP with the highest minor allele frequency which was in strong linkage disequilibrium with the other SNPs within the gene. The number of rare alleles was calculated and categorized into three groups; <3, 3–5, >5. Odds ratios and 95% confidence intervals were calculated for having 3–5 or >5 rare alleles as compared to having <3 alleles (the reference group) in logistic regression adjusted for age, sex, smoking status and dye exposure.

EpiInfo version 3.3.2 (Centers for Disease Control and Prevention, USA) was used to calculate the power for the case-control study. The study was powered (80%) to detect an odds ratio (OR) of 1.5 with a minor allele frequency of 0.3 or an OR of 1.8 with a minor allele frequency of 0.2 significant at the 1% level. Although a large case-control cohort was studied, the study was underpowered to detect effects for SNPs with low minor allele frequencies.

As a guide to interpretation of results in the context of multiple testing, false positive report probability (FPRP) was calculated according to Wacholder et al [22]. The FPRP is the probability that there is no association given a statistically significant finding and is based on the observed significance level, the power to detect an association at that level and the prior probability that the association is real, used to reflect the strength of the prior hypothesis and preceding data. Given the limited number of previous studies based on our SNP set, a moderate prior probability of 1% was used.

## Results

### Study Subjects

There was no difference in sex or age distribution between the case and control populations (Table 1). The majority of subjects were Caucasian with no difference in ethnicity between cases and controls. Histologically 756 patients (98.0%) had transitional cell carcinomas, nine (1.2%) had pure squamous cell carcinomas, three (0.4%) had pure adenocarcinomas and three patients (0.4%) had neuroendocrine, sarcomatoid and leiomyosarcoma respectively. The distribution of stage and grade are shown in Table 1.

**Table 1:** The distribution of sex, age and ethnicity amongst 771 cases and 800 controls and Odds ratios (OR) and 95% Confidence Intervals (95%CI) from nine unadjusted logistic regression models for the effect of smoking and occupational exposures on bladder cancer risk

**Characteristic**	**Categories**	**Statistic**	**Number of Cases (%)**	**Number of Controls (%)**	**OR (95% Cl)**	**p-value**
	Stage of bladder tumour	N (%)				
	Superficial		322 (41.8)			
	Carcinoma in situ		14 (1.8)			
	T1/2		298 (38.7)			
	T3/4		66 (8.5)			
	Unknown		71 (9.2)			
	Grade of bladder tumour	N (%)				
	G1		87 (11.3)			
	G2		313 (40.6)			
	G3		325 (42.1)			
	Unknown		46 (6.0)			
Sex	Male	N (%)	544 (70.9)	527 (67.3)		0.12*
	Female		223 (29.1)	256 (32.7)		
Age		Mean (range)	73.2 (30.1–100.7)	73.1 (28.1–99.7)		0.88**
Ethnicity	Caucasian	N (%)	759 (98.6)	783 (97.9)		
	Non-caucasian		11 (1.4)	17 (2.1)		0.30*
Smoking	Never	N (%)	163 (21.3)	254 (32.5)	1.0	
	Ever		603 (78.7)	528 (67.5)	1.78 (1.42–2.24)	<0.0001
Pack year		Median (range)	21.0 (0–177.9)	11.3 (0–184.0)	1.01 (1.00–1.01)	<0.0001§
Rubber	Ever vs never	N (%)	10 (1.3)	5 (0.6)	2.05 (0.70–6.02)	0.18
Plastics	Ever vs never	N (%)	21 (2.7)	11 (1.4)	1.97 (0.94–4.11)	0.06
Labs	Ever vs never	N (%)	23 (3.0)	14 (1.8)	1.69 (0.86–3.31)	0.11
Printing	Ever vs never	N (%)	45 (5.9)	45 (5.8)	1.01 (0.67–1.56)	0.93
Dyes	Ever vs never	N (%)	56 (7.3)	27 (3.5)	2.20 (1.37–3.52)	0.001
Diesel	Ever vs never	N (%)	88 (11.5)	84 (10.8)	1.07 (0.78–1.42)	0.66
Number of	0	N (%)	569 (74.3)	620 (79.6)	1.0	
occupational	1		165 (21.5)	139 (17.8)	1.27 (1.06–1.51)	0.009§
exposures	2		24 (3.1)	14 (1.8)		
	3		6 (0.8)	5 (0.6)		
	4		0 (0)	1 (0.1)		
	5		0 (0)	0 (0)		
	6		2 (0.3)	0 (0)		

* Pearson's Chi-squared test

** Two-tailed T-test

§ P-value for linear trend

Missing sex for 4 patients, ethnicity for 1 patient and smoking exposures for 5 patient

### Effects of Environmental Risk Factors

Smoking was found to be associated with an increased risk of bladder cancer (OR = 1.78, 95%CI (1.42–2.24) for ever versus never smoked, Table 1). When the data were analysed quantitatively using packyears, a dose response was found with a 1% increase in bladder cancer risk for each packyear smoked (p < 0.0001). Those subjects exposed to occupational carcinogens had an increased bladder cancer risk, the association being strongest for dye exposure (OR = 2.20, 95%CI (1.37–3.52)). When the number of exposures was analysed, there was an estimated 27% increase in bladder cancer risk for each additional occupational exposure (95%CI 1.06–1.51, Table 1). Smoking status (ever versus never) and dye exposure (ever versus never) were both entered into a multivariable model and were found to be independent risk factors for bladder cancer; the adjusted OR for smoking was 1.75 (95%CI 1.38–2.22) and the adjusted OR for dye exposure was 2.15 (95%CI 1.33–3.48) (data not shown).

### Genotyping

All SNPs were successfully genotyped in more than 95% of samples (see Additional file 1). The 5% of samples genotyped in duplicate showed 99.99% concordance. The combined hospital-based and community control genotype distribution did not deviate from Hardy-Weinberg equilibrium for any of the SNPs. However in analysis restricted to the community control group, the genotype distribution for rs643788 deviated from Hardy-Weinberg equilibrium (p = 0.04) and in analysis restricted to the hospital-based control group, the genotype distribution for rs2155209 deviated from Hardy-Weinberg equilibrium (p = 0.01). The minor allele frequencies were consistent with those in the public domain (Table 2). These frequencies were obtained at the start of the study when the frequencies in the dbSNP database described pooled ethnicity. Where the observed minor allele frequency deviated from those in the public domain, this was due to the predominant non-European influence on allele frequencies within the NCBI and Utah databases for that particular SNP. SNPs within each gene were found to be in strong linkage disequilibrium (LD) (Table 3 and Table 4).

**Table 2:** Odds ratios and 95% Confidence Intervals for the effect of each SNP on bladder cancer risk from unadjusted and adjusted logistic regression models (adjusted for age, sex, smoking status and exposure to dyes)

**SNP rs number, function, allele substitution**	Control minor allele frequency (published)*	**No of rare alleles**	**Number of Cases**	**Number of Controls**	**Unadjusted OR (95% CI)**	**p value for un-adjusted OR**	**Adjusted OR (95% CI)**	**p value for adjusted OR**
**NBS1**								

rs1448 3'UTR A/G	0.06 (0.189)	0	670 (89.0)	695 (89.1)	1		1	
		1	81 (10.8)	85 (10.9)	0.99 (0.71–1.36)	0.94	0.98 (0.71–1.37)	0.91
		2	2 (0.3)	0 (0)	ND^a^		ND	
rs9995 3'UTR A/G	0.31 (0.36)	0	334 (44.2)	373 (47.5)	1		1	
		1	347 (46.0)	332 (42.3)	1.17 (0.95–1.44)	0.15	1.16 (0.94–1.44)	0.17
		2	74 (9.8)	80 (10.2)	1.03 (0.73–1.40)	0.86	1.04 (0.73–1.48)	0.85
rs13312986 3'UTR A/G	0.01 (0.067)	0	717 (95.1)	765 (97.3)	1		1	
		1	36 (4.8)	21 (2.7)	1.83 (1.06–3.16)	0.02	1.72 (0.99–3.02)	0.06
		2	1 (0.1)	0 (0)	ND		ND	
rs1063054 3'UTR T/G	0.31 (0.33)	0	329 (44.3)	374 (47.8)	1		1	
		1	340 (45.8)	330 (42.2)	1.17 (0.95–1.45)	0.14	1.15 (0.93–1.44)	0.18
		2	74 (10.0)	79 (10.1)	1.06 (0.75–1.51)	0.73	1.06 (0.75–1.53)	0.71
rs13312981 3'UTR A/G	0.006 (0.034)	0	751 (99.6)	771 (98.7)	1		1	
		1	3 (0.4)	10 (1.3)	0.31 (0.08–1.12)	0.05	0.35 (0.09–1.27)	0.11
		2	0 (0)	0 (0)	ND		ND	
rs2735383 3'UTR G/C	0.32 (0.335)	0	328 (44.6)	363 (47.1)	1		1	
		1	336 (45.7)	326 (42.3)	1.14 (0.92–1.41)	0.23	1.14 (0.92–1.41)	0.27
		2	72 (9.8)	81 (10.5)	0.98 (0.69–1.40)	0.93	0.98 (0.69–1.40)	0.93
rs1063053 3'UTR C/T	0.32 (0.312)	0	331 (44.4)	370 (47.2)	1		1	
		1	340 (45.6)	334 (42.6)	1.14 (0.92–1.41)	0.23	1.13 (0.92–1.41)	0.25
		2	74 (9.9)	80 (10.2)	1.03 (0.72–1.47)	0.85	1.04 (0.73–1.49)	0.83
rs1805794 NS G/C Glu185Gln	0.31 (0.306)	0	347 (46.4)	375 (47.6)	1		1	
		1	332 (44.4)	330 (41.9)	1.08 (0.88–1.34)	0.44	1.06 (0.86–1.32)	0.57
		2	69 (9.2)	83 (10.5)	0.89 (0.63–1.28)	0.55	0.93 (0.65–1.33)	0.68
rs769420 NS G/A Leu266Pro	0.001 (0.033)	0	756 (99.7)	782 (99.7)	1		1	
		1	2 (0.3)	2 (0.3)	1.03 (0.15–7.36)	0.97	1.04 (0.14–7.54)	0.97
		2	0 (0)	0 (0)	ND		ND	

**MRE11**								

rs2155209 3'UTR A/G	0.34 (0.27)	0	306 (41.0)	324 (41.9)	1		1	
		1	325 (43.6)	367 (47.5)	0.93 (0.76–1.16)	0.50	0.90 (0.72–1.12)	0.34
		2	115 (15.4)	82 (10.6)	1.48 (1.07–2.05)	0.02	1.39 (1.00–1.94)	0.05
		2v(0+1)					1.54 (1.13–2.08) ^b^	0.01
rs1061956 3'UTR A/G	0.008 (0.04)	0	747 (98.8)	774 (98.5)	1		1	
		1	9 (1.2)	12 (1.5)	0.78 (0.33–1.86)	0.57	0.71 (0.29–1.72)	0.45
		2	0 (0)	0 (0)	ND		ND	
rs641936 5'UTR A/G	0.31 (0.45)	0	352 (47.9)	364 (47.4)	1		1	
		1	308 (41.9)	329 (42.8)	0.97 (0.78–1.20)	0.77	0.98 (0.79–1.21)	0.85
		2	75 (10.2)	75 (9.8)	1.03 (0.73–1.47)	0.85	1.16 (0.81–1.67)	0.43
rs1805365 5'UTR T/C	0.008 (0.048)	0	727 (98.8)	763 (98.3)	1		1	
		1	9 (1.2)	13 (1.7)	0.73 (0.31–1.71)	0.47	0.66 (0.27–1.58)	0.35
		2	0 (0)	0 (0)	ND		ND	
rs535801 SS C/T	0.29 (0.417)	0	381 (52.1)	393 (51.2)	1		1	
		1	292 (40.0)	307 (40.0)	0.98 (0.79–1.21)	0.86	1.00 (0.81–1.25)	0.97
		2	58 (7.9)	68 (8.9)	0.88 (0.60–1.28)	0.51	1.00 (0.68–1.49)	0.98
rs497763 3'UTR G/A	0.42 (0.488)	0	259 (34.5)	278 (35.4)	1		1	
		1	373 (49.7)	363 (46.2)	1.10 (0.88–1.38)	0.39	1.13 (0.90–1.42)	0.35
		2	119 (15.9)	145 (18.5)	0.88 (0.66–1.18)	0.40	0.97 (0.72–1.32)	0.85

**ATM**								

rs2234997 NS A/T Glu126Asp	0.001 (0.001)	0	744 (99.5)	784 (99.9)	1		1	
		1	4 (0.5)	1 (0.1)	4.22 (0.47–37.8)	0.15	4.20 (0.46–38.1)	0.20
		2	0 (0)	0 (0)	ND		ND	
rs3092856 NS C/T Tyr32His	0.001 (0.035)	0	753 (100)	788 (99.8)	1		1	
		1	0 (0)	2 (0.3)	ND		ND	
		2	0 (0)	0 (0)	ND		ND	
rs582157 3'UTR T/A	0.46 (0.438)	0	215 (28.7)	220 (28.2)	1		1	
		1	380 (50.8)	400 (51.3)	0.97 (0.77–1.23)	0.81	0.98 (0.77–1.24)	0.85
		2	153 (20.5)	160 (20.5)	0.98 (0.73–1.31)	0.88	1.00 (0.75–1.35)	0.96
rs1263936 3'UTR G/A	0.45 (0.375)	0	225 (30.2)	229 (29.5)	1		1	
		1	381 (51.1)	391 (50.3)	0.99 (0.79–1.25)	0.94	0.99 (0.78–1.25)	0.90
		2	140 (18.8)	157 (20.2)	0.91 (0.68–1.22)	0.52	0.94 (0.70–1.27)	0.70
rs609261 5'UTR A/G	0.46 (0.49)	0	215 (28.6)	221 (28.3)	1		1	
		1	384 (51.1)	396 (50.6)	1.00 (0.79–1.26)	0.98	1.00 (0.79–1.27)	0.98
		2	152 (20.2)	165 (21.1)	0.95 (0.71–1.26)	0.71	0.98 (0.73–1.31)	0.88

**RAD50**								

rs1047382 NS A/G Ala558Val	0.001 (0.095)	0	755 (100)	789 (99.9)	1		1	
		1	0 (0)	1 (0.1)	ND		ND	
		2	0 (0)	0 (0)	ND		ND	

**H2AX**								

rs643788 3'UTR A/G	0.42 (0.491)	0	235 (31.8)	272 (35.1)	1		1	
		1	368 (49.8)	354 (45.7)	1.20 (0.96–1.51)	0.11	1.20 (0.95–1.51)	0.13
		2	136 (18.4)	149 (19.2)	1.06 (0.80–1.41)	0.71	1.03 (0.76–1.38)	0.86
rs8551 3'UTR G/A	0.45 (0.485)	0	236 (31.5)	277 (35.4)	1		1	
		1	369 (49.2)	371 (47.4)	1.15 (0.93–1.46)	0.18	1.16 (0.92–1.46)	0.20
		2	145 (19.3)	134 (17.1)	1.27 (0.95–1.70)	0.11	1.25 (0.93–1.69)	0.14
rs7350 INT G/A	0.36 (0.413)	0	286 (38.4)	325 (41.8)	1		1	
		1	353 (47.5)	342 (44.0)	1.17 (0.94–1.46)	0.15	1.20 (0.96–1.50)	0.11
		2	105 (14.1)	111(14.3)	1.07 (0.79–1.47)	0.65	1.03 (0.75–1.41)	0.87

a) ND – Not determined because of zero count for case or control population.

b) The OR was calculated by comparing the rare homozygotes with the combined common homozygotes and heterozygotes as the bladder cancer risk appeared to be confined to the rare homozygotes.

* Allele frequencies obtained from the Environmental Genome Project (EGP) via the National Center for Biotechnology Information (NCBI) at start of study (ie. pooled ethnicity)

**Table 3:** Linkage disequilbrium as determined using Lewontins D' between SNPs in MRE11, ATM and H2AX genes.

		***MRE11***				***ATM***			***H2AX***		
		
		rs 2155209	rs 641936	rs 535801	rs 497763	rs 582157	rs 1263936	rs 609261	rs 643788	rs 8551	rs 7350
***MRE11***	rs 2155209	**0.36 **^1^									
	rs 641936	0.99	**0.31**								
	rs 535801	0.87	0.92	**0.28**							
	rs 497763	0.90	0.88	1.0	**0.41**						
***ATM***	rs 582157	0	0	0	0.01	**0.46**					
	rs 1263936	0.01	0.02	0	0	0.91	**0.45**				
	rs 609261	0.01	0.01	0.01	0.01	0.90	0.97	**0.46**			
***H2AX***	rs 643788	0.01	0.05	0.04	0.01	0.01	0.02	0.02	**0.43**		
	rs 8551	0.01	0.05	0.05	0.01	0.01	0.02	0.02	1.0	**0.42**	
	rs 7350	0.02	0.01	0.01	0.02	0.02	0.01	0.01	0.98	0.94	**0.37**

(1) Numbers in bold along the diagonal of the tables represent the minor allele frequency

**Table 4:** Linkage disequilbrium as determined using Lewontins D' between SNPs in the NBS1 gene.

		**NBS1**						
		
		**rs1448**	**rs9995**	**rs13312986**	**rs1063054**	**rs2735383**	**rs1063053**	**rs1805794**
**NBS1**	**rs1448**	**0.06 **^1^						
	**rs9995**	1.0	**0.32**					
	**rs13312986**	0.98	0.94	**0.02**				
	**rs1063054**	1.0	0.99	0.92	**0.32**			
	**rs2735383**	1.0	0.99	0.95	0.99	**0.32**		
	**rs1063053**	1.0	0.98	0.95	0.99	0.99	**0.32**	
	**rs1805794**	0.25	0.70	0.92	0.71	0.71	0.71	**0.31**

SNPs with a minor allele frequency less than 1% were excluded

(1) Numbers in bold along the diagonal of the tables represent the minor allele frequency

Only one of the 24 SNPs, the *MRE11 *3'UTR SNP rs2155209 showed an association with bladder cancer risk (Table 2). In analyses adjusting for age, sex, smoking and occupational dye exposure, individuals homozygous for the rare allele of rs2155209 had an OR of 1.54 (95% CI 1.13–2.08, p = 0.01) when compared to those carrying the common homozygous genotype or heterozygous genotype. In analysis restricted to the Caucasian subjects the results were very similar (adjusted OR = 1.47, 95%CI (1.08–2.00) p = 0.02). However, the rs2155209 genotype distribution deviated from Hardy-Weinberg equilibrium in the hospital-based control group and when the genotype distribution of the community controls was compared to the cases, there was no evidence of an effect (adjusted OR = 0.96, 95% CI (0.61–1.49) for the rare homozygotes compared to the grouped common homozygotes/heterozygotes). The false positive report probability (FPRP) for the observed association was 53%, so the finding is approximately equally likely to be a true or a false finding. No haplotypes were found to increase bladder cancer risk in any gene (Table 5).

**Table 5:** Haplotype analysis for association with bladder cancer risk

**Gene**	Haplotype (%)*	**OR (95% CI)**	**p-value**
**NBS1**			

	AAATGCG (59.3)	1.0	
	AGAGCTC (25.5)	1.06 (0.90–1.26)	0.49
	AGAGCTG (6.4)	1.13 (0.86–1.49)	0.44
	Rare<5% (8.8)	1.04 (0.88–1.22)	0.55

**MRE11**			

	AACA (34.2)	1.0	
	AACG (26.3)	0.88 (0.68–1.13)	0.32
	AGTA (22.2)	0.83 (0.68–1.01)	0.07
	GACG (10.2)	0.92 (0.76–1.11)	0.38
	Rare<5% (7.1)	0.90 (0.73–1.10)	0.34

**ATM**			

	TGG (52.0)	1.0	
	AAA (43.3)	0.97 (0.84–1.13)	0.73
	Rare<5% (4.7)	1.01 (0.87–1.17)	0.77

**H2AX**			

	AGG (55.9)	1.0	
	GAA (35.5)	1.11 (0.95–1.29)	0.20
	GAG (6.8)	1.13 (0.87–1.46)	0.44
	Rare<5% (1.8)	1.02 (0.86–1.21)	0.61

SNPs with a minor allele frequency less than 1% were excluded

NBS1 SNPs were, in order, rs1448 (A/G) rs9995 (A/G) rs13312986 (A/G) rs1063054 (T/G) rs2735383 (G/C) rs1063053 (C/T) rs1805794 (G/C)

MRE11 SNPs were, in order, rs2155209 (A/G) rs641936 (A/G) rs535801 (C/T) rs497763 (G/A)

ATM SNPs were, in order, rs582157 (T/A) rs1263936 (G/A) rs609261 (A/G)

H2AX SNPs were, in order, rs643788 (A/G) rs8551 (G/A) rs7350 (G/A)

* Percentage frequencies of haplotype(s) are indicated in brackets

### Gene-environment interactions

The effects of smoking status and dye exposure on bladder cancer risk stratified by genotype are shown in Additional files 2 and 3 respectively. There was no suggestion of interaction between smoking status or dye exposure and any variant.

### Analysis of multiple variants in the same pathway

The number of rare alleles was calculated from four SNPs (rs2735383, rs497763, rs609261 and rs8551) to represent the maximum variation within the subject population. No association with bladder cancer risk was found for individuals with increasing numbers of rare alleles when adjusting for age, sex, smoking status and dye exposure (see Additional file 4). When the analysis was repeated using the SNP rs2155209, there was still no effect of number of rare alleles with bladder cancer risk.

### Confirmatory sequencing of MRE11 variant rs2155209

Sixty-seven of the 95 selected samples were successfully genotyped. The failure of the remaining 29 was attributed to the DNA quality and quantity. The wildtype genotype was confirmed in 23 samples and the homozygous variant in 20. Of 23 sequenced samples found to be heterozygous on Taqman genotyping, 20 were definitely confirmed as heterozygous on genotyping and in a further three the C allele was a very minor species.

## Discussion

To our knowledge, this is the first epidemiological study to focus on the DSB signalling pathway in bladder cancer, evaluating the effect of potentially functional variants in *ATM, MRE11, NBS1, RAD50 *and *H2AX *on bladder cancer risk.

We found an association for the *MRE11 *SNP rs2155209 with bladder cancer risk. If a Bonferroni correction had been used to account for multiple testing in this study, no SNP would be significantly associated with bladder cancer. However, a Bonferroni correction is likely to be an overcorrection as Bonferroni assumes independence between multiple tests. We calculated the false positive report probability for the observed association as an alternative method of correcting for multiple testing and found the result to be approximately equally likely to be a true or a false finding. The lack of Hardy-Weinberg equilibrium amongst the hospital-based control group for this SNP may provide some evidence that this result is a false positive, considering that no effect of rs2155209 was seen when comparing the community controls with the cases. Therefore this result requires validation in a further cohort.

In line with the current literature [38-41] we found a strong association between bladder cancer risk and both smoking and dye exposure, and a weaker association with plastics manufacturing. However, there was no modification of these effects when stratifying by SNP genotype.

As MRE11, NBS1, RAD50, ATM, and H2AX interact with each other to facilitate DSB damage signalling, we hypothesized that there may be a gene dosage effect in our study, with subjects with increasing numbers of high risk alleles having an increased risk of bladder cancer. However, there was no difference found between the case and control populations with adjusted odds ratios of ~1.0 and tight confidence intervals.

The case and control populations had similar age and sex distributions with no significant difference in ethnicity. Quality control was stringent in the study with a low proportion of undetermined samples and high concordance among the duplicate samples. The sequencing of MRE11 variant rs2155209 confirmed the Taqman genotyping results. One of the strengths of this study is the detailed information on occupational history in a mainly Caucasian population based in West Yorkshire which allows the investigation of gene-environment interactions. Despite this, it is likely that the study was underpowered to detect such interactions. There is always an inherent recall bias in this sort of questionnaire-based study, with the possibility that case subjects are more likely to remember smoking dose and any hazardous exposures. Occupational exposure was difficult to quantify from the interviews.

The *MRE11 *variant associated with bladder cancer was located in the 3'UTR of the gene. The 3'UTR has been implicated in regulation of transcription and mRNA stability [42]. Variants in this region of a gene may have functional significance by affecting transcription and leading to reduced or abnormal protein expression. Alternatively, the variant may be in linkage disequilibrium with a functional variant nearby.

To our knowledge, there have been no previous studies investigating variants in *ATM, MRE11, RAD50 *or *H2AX *and bladder cancer risk. However, Mongiat-Artus et al have reported mutations of *MRE11 *or *RAD50 *in upper tract urothelial tumours, although numbers are small (four of 58 tumours) [43]. The *MRE11 *3'UTR variant has not yet been investigated for possible cellular functional effects. *In vitro *studies may provide a mechanistic explanation for the increase in bladder cancer risk by determining if the variant leads to reduced transcription or instability of mRNA.

## Conclusion

In conclusion, in this relatively large bladder cancer case-control study, a marginal association with bladder cancer risk was found for the *MRE11 *SNP rs2155209. Associations between bladder cancer risk and both smoking and dye exposure were confirmed. Results of this study need validation in another case-control cohort and a larger population is required to fully investigate possible interactions of variants with smoking and dye exposure.

## Competing interests

The authors declare that they have no competing interests.

## Authors' contributions

AC carried out the molecular genetic studies, performed the statistical analysis and drafted the manuscript. FE helped with the statistical analysis and drafting of the manuscript. MMI participated in discussion regarding statistical analysis. MC performed the Taqman genotyping. RGB critically appraised the manuscript. DTB and AEK conceived of the study, and participated in its design and helped to draft the manuscript. All authors read and approved the final manuscript.

## Pre-publication history

The pre-publication history for this paper can be accessed here:


